# Camrelizumab Plus Nab-paclitaxel in Patients with Previously Treated Advanced Urothelial Carcinoma: A Multicenter Phase II Study

**DOI:** 10.34133/cancomm.0025

**Published:** 2026-04-21

**Authors:** Haifeng Li, Meiting Chen, Riqing Huang, Qixiang Rong, Jing Hao, Qiufan Zheng, Yanhong Su, Ditian Shu, Yue Zhang, Wei Yang, Xuefen Lei, Yuchen Cai, Cong Xue, Xin An, Yanxia Shi

**Affiliations:** ^1^ State Key Laboratory of Oncology in South China, Guangdong Provincial Clinical Research Center for Cancer, Sun Yat-sen University Cancer Center, Guangzhou 510060, P. R. China.; ^2^ Department of Medical Oncology, Sun Yat-sen University Cancer Center, Guangzhou 510060, P. R. China.; ^3^ Zhongshan School of Medicine, Sun Yat-sen University, Guangzhou 510060, P. R. China.; ^4^ Department of Medical Oncology, The Second Affiliated Hospital of Kunming Medical University, Kunming 650101, P. R. China.

## Abstract

**Background:** Immune checkpoint inhibitor (ICI) plus nab-paclitaxel has emerged as a promising strategy for advanced urothelial carcinoma (aUC). This study investigates the efficacy and safety of camrelizumab combined with nab-paclitaxel in patients with aUC who had progressed following first-line treatment. **Methods:** This multicenter, phase II study enrolled patients with aUC who had previously received at least 1 platinum-based therapy. Patients with prior ICI-based therapies were also included. Participants received 200 mg of camrelizumab on day 1 (D1) and 125 mg/m^2^ of nab-paclitaxel on D1 and D8, on a 21-d cycle, until disease progression or unacceptable toxicity. The primary end point was progression-free survival (PFS). Potential biomarkers were identified through targeted gene sequencing and by quantifying pretreatment serum levels of cytokines, chemokines, growth factors, and other soluble proteins. **Results:** From 2020 June 12 to 2024 April 1, a total of 60 eligible patients were enrolled. The median age was 62.5 years, 44 patients (73.33%) were male, 44 patients (73.33%) had upper tract urothelial carcinoma, and 17 patients (28.33%) received ICI-based therapies before recruitment. After a median follow-up of 27.50 months (95% confidence interval [CI], 21.90–not reached [NR]), 47 patients (78.33%) experienced disease progression. The median PFS and overall survival were 4.66 months (95% CI, 4.13 to 8.50 months) and 15.70 months (95% CI, 12.17–NR) in the full analysis set, respectively. In addition, the median PFS and overall survival were 6.45 (95% CI, 4.30 to 9.98) months and 17.98 (95% CI, 15.44–NR) months in the per-protocol set, respectively. The objective response rate was 37.04%, with 4 patients achieving complete responses and 16 patients achieving partial responses. Notably, 15 patients experienced durable responses lasting over 1 year. Adverse events of any grade occurred in 59 patients (98.33%), while grade ≥3 adverse events were reported in 31 patients (51.67%). One patient experienced immune-related pneumonia. Biomarker analyses suggested that the elevated pretreatment serum levels of cluster of differentiation 25, interleukin-6, and insulin-like growth factor binding protein 2 were associated with worse treatment responses. **Conclusions:** Camrelizumab plus nab-paclitaxel demonstrated modest antitumor activity along with manageable toxicity in patients with previously treated aUC. **Trial registration:** This trial was registered at www.chictr.org.cn (Identifier: CTR2000033820, registration date: 2020 June 13).

## Background

Urothelial carcinoma (UC) is one of the most common cancers in the world. In China, UC ranks as the 11th most common malignancy [1]. In recent years, the therapeutic landscape of UC has evolved substantially since the advent of immune checkpoint inhibitors (ICIs). ICIs have become a cornerstone of treatment across multiple disease stages [2]. Current guidelines recommend enfortumab vedotin (EV) plus pembrolizumab or platinum-based chemotherapy combined with nivolumab as the preferred first-line treatment for patients with locally advanced or metastatic disease, rather than platinum-based chemotherapy monotherapy [3,4].

For patients who experience relapse or progression after first-line therapy, ICI monotherapy is recommended according to current guidelines. However, the clinical benefits of ICI monotherapy remain unsatisfactory, with median progression-free survival (PFS) ranging from 1.5 to 2.1 months and median overall survival (OS) ranging from 7.0 to 11.1 months [2]. Consequently, the prognosis for patients with advanced disease remains poor, suggesting the need for more effective therapeutic strategies. Prior to the ICI era, nonplatinum agents such as taxanes or vinflunine were the standard of care in the second-line setting [5,6]. More recently, nanoparticle albumin-bound formulation of paclitaxel (nab-paclitaxel) has demonstrated antitumor activity in platinum-refractory advanced urothelial carcinoma (aUC) [7]. Moreover, nab-paclitaxel has shown enhanced efficacy when combined with ICIs in various solid tumors compared with nab-paclitaxel monotherapy [8–10].

Over the past several years, multiple clinical trials have investigated the efficacy of nab-paclitaxel combined with ICIs in aUC [11–13]. In the PEANUT phase II study, pembrolizumab plus nab-paclitaxel was used as salvage therapy in platinum-treated aUC and provided a median PFS of approximately 5 to 6 months (updated analyses reported around 5.1 months) [11,14]. Consistently, another phase II study found that the same combination therapy led to a median PFS of 6.8 months. However, nearly half of the patients in that study were treated in the first-line setting [12]. Notably, participants in these studies were ICI-naïve, and primary tumors were predominantly of bladder origin. In China, upper tract UC (UTUC) accounts for 20% to 30% of all UC cases [1]. Consequently, the current evidence may not fully reflect treatment efficacy in contemporary real-world clinical practice, particularly among patients with UTUC or those receiving ICIs.

Given this context and the limitations of existing evidence, there is an urgent need to improve outcomes in patients with aUC who progressed after first-line treatment. Here, we report the efficacy and safety of camrelizumab plus nab-paclitaxel in patients with platinum-resistant aUC receiving this regimen as later-line treatment.

## Materials and Methods

### Study design and patient eligibility

This was a single-arm, phase II, multicenter study in which patients were enrolled between 2020 June 12 and 2024 April 1, evaluating camrelizumab plus nab-paclitaxel in platinum-resistant patients with unresectable locally advanced or metastatic UC. The trial was conducted at 2 centers in China (Sun Yat-sen University Cancer Center, Guangzhou, and the Second Affiliated Hospital of Kunming Medical University, Kunming) and is registered at chinadrugtrials.org.cn (CTR2000033820).

Eligible patients were required to be ≥18 years old with a diagnosis of aUC, an Eastern Cooperative Oncology Group performance status of 0 to 2, and creatinine clearance of ≥30 ml/min. All patients had received at least 1 systemic therapy for advanced disease, including a platinum-based regimen. Neoadjuvant or adjuvant therapies were considered as prior treatments if disease progression or relapse occurred within 6 months of completion. Prior ICI treatment was permitted. Key exclusion criteria included prior taxane treatment, active autoimmune diseases, or receiving steroid treatment with an equivalent of >10 mg of prednisone for any condition. The inclusion and exclusion criteria are further detailed in the Supplementary Materials.

### Intervention

Patients received 200 mg of camrelizumab on day 1 (D1) and 125 mg/m^2^ of nab-paclitaxel on D1 and D8, on a 21-d cycle, until disease progression or unacceptable toxicity. Stepwise doses of nab-paclitaxel were 100 and 75 mg/m^2^ based on toxicities. Nab-paclitaxel discontinuation was allowed after 6 cycles per patient or at the investigator’s discretion. The single agent could be continued alone if the other agent was discontinued due to toxicity. The maximum duration of camrelizumab administration was 24 months. Radiological assessments were performed every 2 cycles, and tumor responses were evaluated according to the Response Evaluation Criteria in Solid Tumors version 1.1 [15]. Adverse events (AEs) were graded according to the Common Terminology Criteria for Adverse Events classification (version 5.0). The primary end point was PFS. Secondary end points were OS, objective response rate (ORR), disease control rate (DCR), and safety.

The study protocol was reviewed and approved by the ethical committees of each participating center, including Sun Yat-sen University Cancer Center and the Second Affiliated Hospital of Kunming Medical University. Informed consent was obtained from all participants prior to enrollment, with the option to withdraw at any time for any reason. The study complied with the principles of the Declaration of Helsinki and the Good Clinical Practice guidelines defined by the International Council for Harmonization.

### Biomarker analyses

Programmed cell death ligand 1 (PD-L1) expression was determined by immunohistochemistry using the Ventana SP263 assay, with results reported as tumor proportion score; patients were stratified using a 1% cutoff for further analyses. Targeted gene sequencing (TGS) was performed for patients with available pretreatment tumor specimens. In brief, at least 50 ng of DNA was extracted from formalin-fixed paraffin-embedded tissue specimen. TGS used a pan-cancer panel that includes 1,021 genes (Cat. #DC204802, Geneplus-Beijing). The list of genes covered by this panel is provided in Table S1. Somatic variants meeting the minimum sequencing thresholds of 500× and >5% variant allele fraction were included in further analysis. For fibroblast growth factor receptor 2/3 (*FGFR2/3*), the panel provided full coverage of all coding exons and selected intronic, promoter, and fusion breakpoint regions to detect mutations (single-nucleotide variants and small insertions and deletions), fusions, and copy number variations. The concentrations of a custom panel of 28 serum biomarkers (including cytokines, chemokines, growth factors, and other soluble proteins) were measured in serum samples using Luminex xMAP technology, which is a multiplex bead-based assay system that enables simultaneous detection of multiple analytes [16]. The assay was performed according to the manufacturer’s protocol of Bio-Plex Pro Human Cytokine Assay (Cat. #APT-P20240700346, Bio-Rad).

### Statistical analyses

The study was designed to detect an improvement in median PFS from 2.0 months under the null hypothesis (H₀) to 3.0 months under the alternative hypothesis (H₁). With a 2-sided type I error of 5% and 80% power, assuming exponential survival, a minimum follow-up of 12 months after the end of accrual, and an anticipated dropout rate of 10%, a total of 60 patients were required. PFS and OS were estimated using the Kaplan–Meier method, and median values with 95% confidence intervals (CIs) were reported. ORR, DCR, and safety analyses were based on descriptive statistical summaries. Subgroup analyses were performed using Fisher’s exact test for categorical variables and the Wilcoxon test for continuous variables. Cox regression models were used to analyze the association between baseline characteristics and survival outcomes (PFS and OS). A 2-sided *P* value less than 0.05 was considered statistically significant for all analyses. All statistical analyses were performed using R software (version 4.02).

## Results

### Patient baseline characteristics

**Fig. 1.**
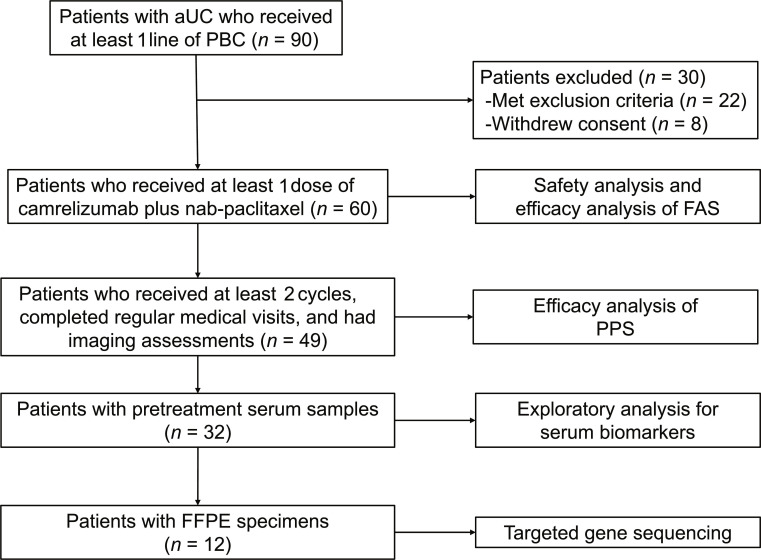
Study workflow. A total of 90 patients were screened for eligibility, and 60 patients were enrolled. All enrolled patients were included in the FAS. Patients who completed regular medical visits and had imaging assessments, received at least 2 treatment cycles, and did not withdraw consent were included in PPS. aUC, advanced urothelial carcinoma; FAS, full analysis set; FFPE, formalin-fixed paraffin-embedded; PBC, platinum-based chemotherapy; PPS, per-protocol set.

Between 2020 June 12 and 2024 April 1, 90 patients were screened, and 60 patients were enrolled (Fig. 1). Baseline characteristics of participants are summarized in Table 1. Briefly, the median age was 62.5 (interquartile range, 55.0 to 67.0) years, 44 patients (73.33%) were male, 7 patients (11.67%) had an Eastern Cooperative Oncology Group performance status of 2, 44 patients (73.33%) had tumors of upper tract origin, and 58 patients (96.67%) had metastases with a mean number of metastatic sites of 1.8 (range, 1 to 5). A total of 17 patients (28.33%) had received prior ICI-based therapy, of whom 7 received it as first-line treatment. Meanwhile, 13 patients (21.67%) received more than 1 line of systemic therapy for advanced disease, including ICI-based regimens (*n* = 10), human epidermal growth factor receptor 2-directed antibody–drug conjugate (*n* = 3), and multiple antigen-stimulating cell therapy (*n* = 2).

**Table 1.** Patient baseline characteristics (*n* = 60)

Characteristics	*n* (%)
Age, median (IQR), years	62.5 (55.0–67.0)
<65	33 (55.00%)
≥65	27 (45.00%)
Sex	
Female	16 (26.67%)
Male	44 (73.33%)
ECOG PS	
0	17 (28.33%)
1	36 (60.00%)
2	7 (11.67%)
Site of primary tumor	
BUC	16 (26.67%)
UTUC	44 (73.33%)
Pathology	
UC NOS	55 (91.67%)
UC and glandular component	1 (1.67%)
UC and squamous cell carcinoma component	3 (5.00%)
Other variants	1 (1.67%)
Metastases	58 (96.67%)
Number of metastatic sites, mean (range)	1.8 (1–5)
Site of metastases	
Lymph nodes only	15 (25.00%)
Bone metastases	1 (1.67%)
Liver metastases	3 (5.00%)
Lung metastases	7 (11.67%)
Peritoneal metastases	2 (3.33%)
Multivisceral metastases	30 (50.00%)
Not available	2 (3.33%)
PD-L1 status	
<1%	23 (38.33%)
≥1%	5 (8.33%)
Not available	32 (53.33%)
Number of previous systemic regimens	
1	47 (78.33%)
>1	13 (21.67%)
Previous ICI-based therapy	17 (28.33%)

BUC, bladder urothelial carcinoma; ECOG PS, Eastern Cooperative Oncology Group performance status; ICI, immune checkpoint inhibitor; IQR, interquartile range; NOS, not otherwise specified; PD-L1, programmed cell death ligand 1; UC, urothelial carcinoma; UTUC, upper tract urothelial carcinoma

### Safety

Patients received a median of 10 treatment cycles (range, 0.5 to 34), and all the patients received at least 1 dose of treatment. All patients were included in the safety analysis. Nearly all the patients (*n* = 59, 98.33%) experienced AEs of any grade, with 31 patients (51.67%) reporting grade 3 to 4 AEs (Table 2). The most frequent AE of any grade was leukopenia, occurring in 33 patients (55.00%), with neutropenia observed in most cases (*n* = 31, 51.67%). Alopecia (*n* = 29, 48.33%) and peripheral neuropathy (*n* = 29, 48.33%) were second to leukopenia in AEs of any grade, followed by anemia (*n* = 24, 40.00%) and anorexia (*n* = 24, 40.00%). Reactive cutaneous capillary endothelial proliferation related to camrelizumab occurred in 21 patients (35.00%). One patient discontinued treatment due to immune-related pneumonia. Three patients experienced dose adjustment: 2 patients due to grade 3 peripheral sensory neuropathy and 1 patient due to grade 3 leukopenia.

**Table 2.** Safety summary

Treatment-related AEs, *n* (%)	Any grade	Grades 1–2	Grades 3–4
Any AEs	59 (98.33)	58 (96.67)	31 (51.67)
Leukopenia	33 (55.00)	16 (26.67)	17 (28.33)
Neutropenia	31 (51.67)	11 (18.33)	20 (33.33)
Alopecia	29 (48.33)	29 (48.33)	0 (0.00)
Peripheral neuropathy	29 (48.33)	27 (45.00)	2 (3.33)
Anemia	24 (40.00)	15 (25.00)	9 (15.00)
Anorexia	24 (40.00)	23 (38.33)	1 (1.67)
Fatigue	23 (38.33)	22 (36.67)	1 (1.67)
Reactive cutaneous capillary endothelial proliferation	21 (35.00)	21 (35.00)	0 (0.00)
Itching	20 (33.33)	20 (33.33)	0 (0.00)
Nausea vomiting	16 (26.67)	16 (26.67)	0 (0.00)
Constipation	13 (21.67)	13 (21.67)	0 (0.00)
AST increase	10 (16.67)	10 (16.67)	0 (0.00)
Skin rash	10 (16.67)	10 (16.67)	0 (0.00)
ALT increase	9 (15.00)	9 (15.00)	0 (0.00)
Fever	8 (13.33)	7 (11.67)	1 (1.67)
Hypothyroidism	7 (11.67)	7 (11.67)	0 (0.00)
Diarrhea	6 (10.00)	6 (10.00)	0 (0.00)
Infections	6 (10.00)	4 (6.67)	2 (3.33)
Edema	5 (8.33)	5 (8.33)	0 (0.00)
Stomatitis	5 (8.33)	5 (8.33)	0 (0.00)
Pneumonia	4 (6.67)	1 (1.67)	3 (5.00)
Infusion reaction	3 (5.00)	3 (5.00)	0 (0.00)
Febrile neutropenia	2 (3.33)	0 (0.00)	2 (3.33)
Hyperthyroidism	2 (3.33)	2 (3.33)	0 (0.00)
Thrombocytopenia	2 (3.33)	2 (3.33)	0 (0.00)

AE, adverse event; ALT, alanine transaminase; AST, aspartate aminotransferase

### Efficacy

As of the data cutoff date of 2025 July 1, the median follow-up was 27.50 months (95% CI, 21.90–not reached [NR]). All patients who received at least 1 dose (*n* = 60) were included in the full analysis set (FAS). The per-protocol set (PPS) comprised 49 patients who completed regular medical visits and had imaging assessments, received at least 2 treatment cycles, and did not withdraw consent (Fig. 1).

**Fig. 2.**
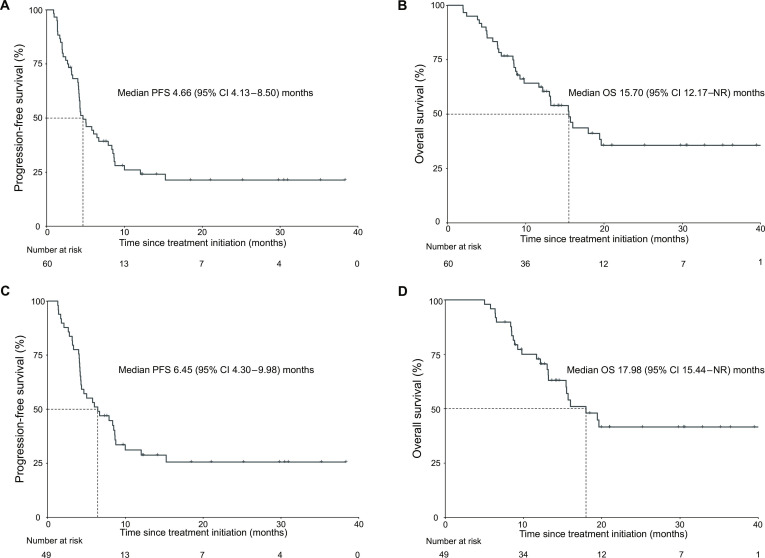
Survival analyses based on FAS and PPS. (A) Kaplan–Meier estimate of progression-free survival in the FAS. (B) Kaplan–Meier estimate of overall survival in the FAS. (C) Kaplan–Meier estimate of progression-free survival in the PPS. (D) Kaplan–Meier estimate of overall survival in the PPS. CI, confidence interval; FAS, full analysis set; NR, not reached; OS, overall survival; PFS, progression-free survival; PPS, per-protocol set.

In the FAS population, the median PFS was 4.66 (95% CI, 4.13 to 8.50) months, and the median OS was 15.70 (95% CI, 12.17–NR) months (Fig. 2A and B). Among 54 FAS patients who underwent imaging assessments, the ORR was 37.04% (95% CI, 24.29% to 51.26%), including 4 complete responses (CRs, 7.41%; 95% CI, 2.06% to 17.89%) and 16 partial responses (29.63%; 95% CI, 17.98% to 43.69%). In addition, 17 patients achieved stable disease (31.48%; 95% CI, 19.52% to 45.56%), resulting in a DCR of 68.52% (95% CI, 54.45% to 80.48%) (Table 3). Meanwhile, in the PPS population, the median PFS was 6.45 (95% CI, 4.30 to 9.98) months, and the median OS was 17.98 (95% CI, 15.44–NR) months (Fig. 2C and D). Among the 49 patients in the PPS population, the ORR and DCR were 40.81% and 73.46%, respectively, including 4 CRs (8.16%), 16 partial responses (32.65%), and 16 stable diseases (32.65%) (Table S2).

**Table 3.** Treatment responses based on RECIST v1.1 in FAS patients with imaging assessments (*n* = 54)

Characteristics	*n*	CR	PR	SD	PD	*P* value
Total	54	4 (7.41%)	16 (29.63%)	17 (31.48%)	17 (31.48%)	
Age						0.106
<65	29	4 (13.79%)	6 (20.69%)	8 (27.59%)	11 (37.93%)	
≥65	25	0 (0.00%)	10 (40.00%)	9 (36.00%)	6 (24.00%)	
Sex						0.190
Female	16	3 (18.75%)	5 (31.25%)	5 (31.25%)	3 (18.75%)	
Male	38	1 (2.63%)	11 (28.95%)	12 (31.58%)	14 (36.84%)	
ECOG PS						0.305
0	14	2 (14.29%)	7 (50.00%)	2 (14.29%)	3 (21.43%)	
1	34	2 (5.88%)	8 (23.53%)	13 (38.24%)	11 (32.35%)	
2	6	0 (0.00%)	1 (16.67%)	2 (33.33%)	3 (50.00%)	
Site of primary tumor						0.682
BUC	13	1 (7.69%)	3 (23.08%)	6 (46.15%)	3 (23.08%)	
UTUC	41	3 (7.32%)	13 (31.71%)	11 (26.83%)	14 (34.15%)	
Pathology						0.318
UC NOS	50	4 (8.00%)	16 (32.00%)	13 (26.00%)	17 (34.00%)	
Other variants	1	0 (0.00%)	0 (0.00%)	1 (100.00%)	0 (0.00%)	
UC and glandular component	1	0 (0.00%)	0 (0.00%)	1 (100.00%)	0 (0.00%)	
UC and squamous cell carcinoma component	2	0 (0.00%)	0 (0.00%)	2 (100.00%)	0 (0.00%)	
Metastases						0.802
Locally advanced	2	0 (0.00%)	1 (50.00%)	1 (50.00%)	0 (0.00%)	
Visceral disease	52	4 (7.69%)	15 (28.85%)	16 (30.77%)	17 (32.69%)	
Site of metastases						0.933
Lymph node only	15	1 (6.67%)	4 (26.67%)	4 (26.67%)	6 (40.00%)	
Liver metastases	3	0 (0.00%)	1 (33.33%)	2 (66.67%)	0 (0.00%)	
Lung metastases	7	1 (14.29%)	2 (28.57%)	3 (42.86%)	1 (14.29%)	
Peritoneal metastases	2	0 (0.00%)	1 (50.00%)	0 (0.00%)	1 (50.00%)	
Multivisceral metastases	25	2 (8.00%)	7 (28.00%)	7 (28.00%)	9 (36.00%)	
PD-L1 status						0.090
<1%	23	0 (0.00%)	4 (17.39%)	10 (43.48%)	9 (39.13%)	
≥1%	5	0 (0.00%)	3 (60.00%)	2 (40.00%)	0 (0.00%)	
Number of previous systemic regimens						0.163
1	41	4 (9.76%)	12 (29.27%)	15 (36.59%)	10 (24.39%)	
>1	13	0 (0.00%)	4 (30.77%)	2 (15.38%)	7 (53.85%)	
Previous ICI-based therapy						0.020
No	38	4 (10.53%)	13 (34.21%)	14 (36.84%)	7 (18.42%)	
Yes	16	0 (0.00%)	3 (18.75%)	3 (18.75%)	10 (62.50%)	

BUC, bladder urothelial carcinoma; CR, complete response; ECOG PS, Eastern Cooperative Oncology Group performance status; FAS, full analysis set; ICI, immune checkpoint inhibitor; NOS, not otherwise specified; PD, progression disease; PR, partial response; PD-L1, programmed cell death ligand 1; RECIST v1.1, Response Evaluation Criteria in Solid Tumors version 1.1; SD, stable disease; UC, urothelial carcinoma; UTUC, upper tract urothelial carcinoma.

**Fig. 3.**
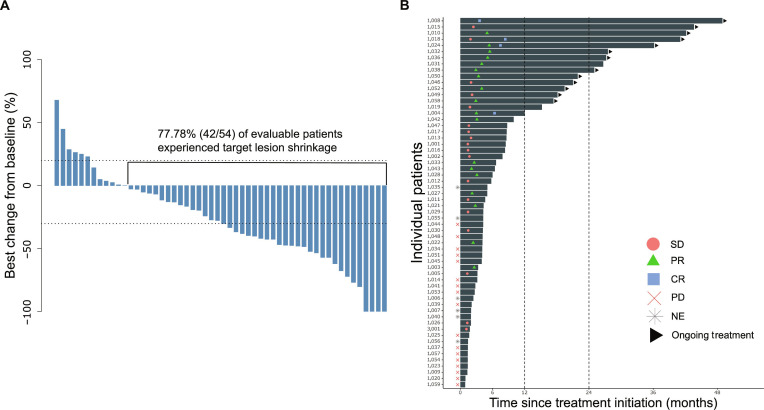
Tumor responses among patients based on the investigator’s assessment. (A) Waterfall plot of the best percentage of change from baseline in the sum of the diameters of target lesions as identified according to RECIST v1.1. Target lesions were reduced in 77.78% (42/54) of patients. (B) The swimmer plot shows the individual patient’s journey from treatment initiation to disease progression. The length of the bar indicates the duration from treatment initiation to disease progression. Tumor response at each assessment is shown as a blue square (complete response), green triangle (partial response), red circle (stable disease), red cross (progression disease), or gray star (not evaluable). The right arrow indicates ongoing treatment. The dashed vertical lines showed that 27.78% (15/54) of patients had durable responses over 12 months and 16.67% (9/54) over 24 months. CR, complete response; NE, not evaluable; PD, progression disease; PR, partial response; RECIST v1.1, Response Evaluation Criteria in Solid Tumors version 1.1; SD, stable disease.

Target lesion shrinkage occurred in 77.78% (42/54) of evaluable patients (Fig. 3A). Additionally, 27.78% patients (15/54) had an ongoing response lasting more than 12 months, and 16.67% patients (9/54) had a durable response lasting over 2 years (Fig. 3B).

### Subgroup analyses

**Fig. 4.**
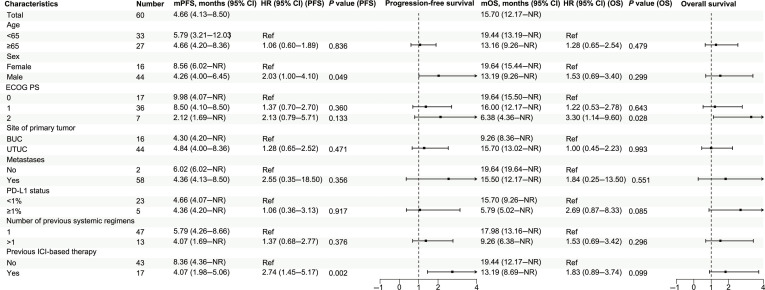
Subgroup analyses of PFS and OS using univariable Cox regression for the FAS population. BUC, bladder urothelial carcinoma; CI, confidence interval; ECOG PS, Eastern Cooperative Oncology Group performance status; FAS, full analysis set; HR, hazard ratio; NR, not reached; ICI, immune checkpoint inhibitor; PD-L1, programmed cell death ligand 1; PFS, progression-free survival; mPFS, median progression-free survival; Ref, reference; OS, overall survival; mOS, median overall survival; UTUC, upper tract urothelial carcinoma.

In the FAS population, univariable and multivariable Cox regression analyses based on baseline characteristics are presented in Fig. 4 and Fig. S1. In the univariable analyses (Fig. 4), no significant differences in PFS or OS were observed between UTUC and BUC (bladder urothelial carcinoma) (PFS: 4.84 months versus 4.30 months, hazard ratio [HR] = 1.28, 95% CI, 0.62 to 2.52; *P* = 0.471; OS: 15.70 months versus 9.26 months, HR = 1.00, 95% CI, 0.45 to 2.23; *P* = 0.993). For patients who previously received ICI-based therapy, there was an association with inferior PFS (HR = 2.74, 95% CI, 1.45 to 5.17; *P* = 0.002) compared with ICI-naïve patients but not with OS (HR = 1.83, 95% CI, 0.89 to 3.74; *P* = 0.099). The median PFS and OS were 4.07 (95% CI, 1.98 to 5.06) months and 13.19 (95% CI, 8.69–NR) months for prior-ICI patients, respectively, as well as 8.36 (95% CI, 4.36–NR) months and 19.44 (95% CI, 12.17–NR) months for ICI-naïve patients, respectively. Male patients had inferior PFS compared with female patients but not OS (PFS: 4.26 months versus 8.56 months, HR = 2.03, 95% CI, 1.00 to 4.10; *P* = 0.049; OS: 13.19 months versus 19.64 months, HR = 1.53, 95% CI, 0.69 to 3.40; *P* = 0.299). Patients treated in a second-line setting showed a trend toward inferior PFS and OS, but the differences were not statistically significant. Among 28 patients with PD-L1 IHC data, there was no statistically significant difference in PFS or OS between ≥1% expression and <1% expression (≥1% versus <1%, PFS: 4.36 months versus 4.66 months, HR = 1.06, 95% CI, 0.36 to 3.13; *P* = 0.917; OS: 5.79 months versus 15.70 months, HR = 2.69, 95% CI, 0.87 to 8.33; *P* = 0.085). In multivariable analyses (Fig. S1), patients with prior ICI-based therapies showed inferior PFS (HR = 2.50, 95% CI, 1.21 to 5.20; *P* = 0.013) but not OS (HR = 1.40, 95% CI, 0.58 to 3.20; *P* = 0.469). Similarly, male patients had inferior PFS (HR = 2.70, 95% CI, 1.27 to 5.90; *P* = 0.010) but not OS (HR = 2.00, 95% CI, 0.82 to 4.80; *P* = 0.129). No statistically significant difference was observed between UTUC and BUC in PFS (HR = 1.90, 95% CI, 0.85 to 4.40; *P* = 0.116) and OS (HR = 1.40, 95% CI, 0.58 to 3.50; *P* = 0.437). Additionally, there was no significant interaction between prior ICI and the site of primary tumor in the multivariable model (*P* for interaction = 0.562).

In the PPS, patients with prior ICI-based therapies had inferior PFS (ICI-pretreated versus ICI-naïve: 4.10 months versus 8.64 months, HR = 3.21, 95% CI, 1.55 to 6.65; *P* = 0.002) but not OS (15.44 months versus NR, HR = 2.14, 95% CI, 0.93 to 4.93; *P* = 0.074). No statistically significant differences were observed in the subgroups of primary tumor (UTUC versus BUC, PFS: 6.35 months versus 6.45 months; OS: 19.44 months versus NR), sex (male versus female, PFS: 4.51 months versus 8.63 months; OS: 16.00 months versus 19.64 months), and PD-L1 status (≥1% versus <1%, PFS: 4.69 months versus 5.22 months; OS: 8.98 months versus 15.70 months) (Fig. S2).

In the patients who were treated in the second-line setting (*n* = 47), male patients had inferior PFS (male versus female, 4.30 months versus 10.33 months, HR = 2.68, 95% CI, 1.17 to 6.14; *P* = 0.019) but not OS (15.50 months versus 19.64 months, HR = 1.56, 95% CI, 0.62 to 3.92; *P* = 0.346). The ICI-pretreated patients had inferior PFS (ICI-pretreated versus ICI-naïve, 4.10 months versus 7.90 months, HR = 3.07, 95% CI, 1.27 to 7.44; *P* = 0.013) but not OS (16.00 months versus 19.44 months, HR = 1.37, 95% CI, 0.51 to 3.65; *P* = 0.533). PD-L1 expression ≥1% was associated with inferior OS (≥1% versus <1%, 8.60 months versus 16.00 months, HR = 7.10, 95% CI, 1.29 to 39.18; *P* = 0.025) but not PFS (4.69 months versus 5.90 months, HR = 1.91, 95% CI, 0.40 to 9.03; *P* = 0.416) (Fig. S3).

### Exploratory analyses

**Fig. 5.**
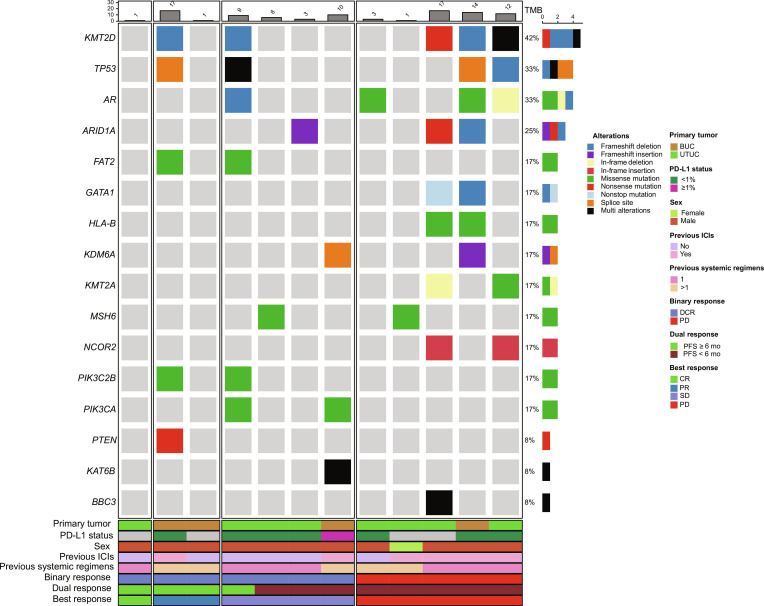
Genetic analyses using TGS (*n* = 12). Oncoplot of a total of 12 available patients, along with tumor responses and baseline characteristics. *AR*, androgen receptor; *ARID1A*, AT-rich interaction domain 1A; *BBC3*, BCL2 binding component 3; BUC, bladder urothelial carcinoma; CR, complete response; DCR, disease control rate; *FAT2*, FAT atypical cadherin 2; *GATA1*, GATA binding protein 1; *HLA-B*, major histocompatibility complex, class I, B; ICI, immune checkpoint inhibitor; *KAT6B*, lysine acetyltransferase 6B; *KDM6A*, lysine demethylase 6A; *KMT2A*, lysine methyltransferase 2A; *KMT2D*, lysine methyltransferase 2D; *MSH6*, mutS homolog 6; *NCOR2*, nuclear receptor corepressor 2; PD, progression disease; PD-L1, programmed cell death ligand 1; PFS, progression-free survival; *PIK3CA*, phosphatidylinositol-4,5-bisphosphate 3-kinase catalytic subunit alpha; *PIK3C2B*, phosphatidylinositol-4-phosphate 3-kinase catalytic subunit type 2 beta; PR, partial response; *PTEN*, phosphatase and tensin homolog; SD, stable disease; TGS, targeted gene sequencing; *TP53*, tumor protein p53; UTUC, upper tract urothelial carcinoma.

Twelve patients provided pretreatment formalin-fixed paraffin-embedded specimens for TGS. The most frequent genomic alterations were observed in lysine methyltransferase 2D (*KMT2D*), tumor protein p53 (*TP53*), and androgen receptor (*AR*) (occurring in >30% of patients) (Fig. 5). No *FGFR2* or *FGFR3* alterations were detected. Based on the duration of response, patients with PFS ≥6 months were classified as responders, and those with PFS <6 months were classified as nonresponders. Phosphatidylinositol-4-phosphate 3-kinase catalytic subunit type 2 beta (*PIK3C2B*), FAT atypical cadherin 2 (*FAT2*), and phosphatidylinositol-4,5-bisphosphate 3-kinase catalytic subunit alpha (*PIK3CA*) were altered only in responders. *AR*, AT-rich interaction domain 1A (*ARID1A*), lysine methyltransferase 2A (*KMT2A*), GATA binding protein 1 (*GATA1*), major histocompatibility complex, class I, B (*HLA-B*), and nuclear receptor corepressor 2 (*NCOR2*) were more frequently altered in nonresponders. However, TGS did not identify significant differences in alteration rates between responders and nonresponders or among other subgroups. Notably, tumor mutational burden was lower in responders than in nonresponders.

**Fig. 6.**
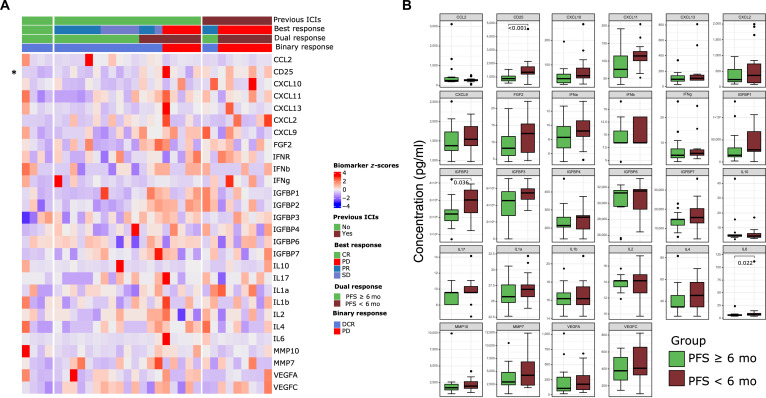
Analyses of pretreatment serum biomarker levels (*n* = 32). (A) Heatmap of total pretreatment serum biomarkers shown without clustering method, * indicates a statistical difference between the ICI-pretreatment subgroup and the ICI-naïve subgroup. (B) The differences between responders (PFS ≥6 months) and nonresponders (PFS <6 months) in pretreatment levels of serum biomarkers, the statistical significances were calculated with the Wilcox test. CR, complete response; DCR, disease control rate; ICI, immune checkpoint inhibitor; PD, progression disease; PR, partial response; PFS, progression-free survival; SD, stable disease.

Pretreatment serum biomarker profiling was conducted in 32 patients. Among them, 23 patients were ICI-naïve, and 9 patients were ICI-pretreated. The level of cluster of differentiation 25 (CD25) in the ICI-naïve group was significantly lower than in the ICI-pretreated group (1,158.74 versus 1,538.56 pg/ml, *P* = 0.011), with no differences in other biomarkers (Fig. 6A). Higher pretreatment levels of CD25, insulin-like growth factor binding protein 2 (IGFBP2), and interleukin-6 (IL-6) were significantly associated with poor response. Mean CD25 was 936.60 pg/ml in responders versus 1,638.40 pg/ml in nonresponders (*P* < 0.001); IGFBP2 was 224,400 pg/ml in responders versus 288,000 pg/ml in nonresponders (*P* = 0.036); and IL-6 was 6.50 pg/ml in responders versus 14.70 pg/ml in nonresponders (*P* = 0.022) (Fig. 6B). For ICI-naïve patients, responders had significantly lower levels of CD25, C-X-C motif chemokine ligand 9 (CXCL9), CXCL11, IGFBP1, IGFBP2, IL-4, IL-6, and matrix metallopeptidase 10 compared with nonresponders (all *P* < 0.05). Only 9 patients were ICI-pretreated (2 responders and 7 nonresponders), and no statistically significant differences were observed (Fig. S4 and Table S3). In the survival analyses, using median serum biomarker levels as cutoffs, low CD25 was significantly associated with superior PFS (Low versus High; HR = 0.26, 95% CI, 0.11 to 0.63; *P* = 0.003) and OS (Low versus High; HR = 0.25, 95% CI, 0.09 to 0.75; *P* = 0.013). Similarly, low-pretreatment CXCL9 and IL-4 were significantly associated with superior PFS ([HR = 0.43, 95% CI, 0.18 to 0.99; *P* = 0.049] and [HR = 0.44, 95% CI, 0.19 to 0.99; *P* = 0.047], respectively). In addition, low-pretreatment IGFBP1 was significantly associated with superior OS (HR = 0.25, 95% CI, 0.08 to 0.78; *P* = 0.017) (Fig. S5).

## Discussion

In the present study, camrelizumab combined with nab-paclitaxel demonstrated clinically meaningful activity in patients with platinum-resistant aUC. In the FAS, the combination achieved a median PFS of 4.66 months and a median OS of 15.70 months. In the PPS, median PFS and OS were 6.45 and 17.98 months, respectively. These results align with other trials evaluating nab-paclitaxel plus ICIs in later-line settings [11–13]. Compared with conventional later-line therapies such as docetaxel, paclitaxel, or vinflunine, the camrelizumab plus nab-paclitaxel regimen conferred approximately 2 months of improvement in PFS and 9 months in OS [17]. Notably, the median OS of 13.19 months observed in patients with prior ICI therapy appears comparable to the median OS reported for later-line antibody–drug conjugate monotherapy, such as EV in the EV-301 trial (median OS of 12.91 months) and sacituzumab govitecan in the TROPiCS-04 trial (median OS of 10.30 months) [18,19]. However, this finding is based on a small cohort of only 17 patients, which may limit its reliability. The toxicity profile was manageable, with no grade 5 AE reported. Importantly, patients previously exposed to ICI-based therapies still derived meaningful benefit from camrelizumab plus nab-paclitaxel. Therefore, our study provides further support for combining ICIs with nonplatinum agents in patients with advanced disease.

UTUC, which constitutes only 5% to 10% of UC cases worldwide, exhibits distinct clinicopathologic and genomic features compared with BUC, including differences in tumor grading, genomic alterations, and disease heterogeneity [20]. Patients with advanced UTUC usually present with high tumor burden and impaired renal function. Previous clinical trials involving ICIs have underrepresented UTUC, with recruitment percentages of only around 20%, potentially compromising efficacy outcomes [11–14]. In the present study, 44 patients with UTUC were enrolled, accounting for 73.33% of the total cohort, with a mean of 1.8 metastatic sites. Notably, in the present study, the UTUC group demonstrated comparable short-term response, PFS, and OS compared with the BUC group, providing further support for the use of ICIs in UTUC.

In contemporary clinical practice, ICI-based regimens are recommended as first-line treatment for aUC, meaning that patients who progress after initial treatment are usually classified as ICI-pretreated. Previous evidence for nab-paclitaxel combined with ICI in aUC has largely been restricted to ICI-naïve patients [12,14]. For example, the PEANUT study (pembrolizumab plus nab-paclitaxel) only recruited patients who were both taxanes- and ICI-naïve, and reported a median PFS of 5.1 months in updated analyses [11,14]. In an American study of a similar combination, half of the patients were treatment-naïve and reported a PFS of 6.8 months [12]. In another study, nab-paclitaxel plus socazolimab was investigated in treatment-naïve patients with a PFS of 8.3 months [13]. In the present study, ICI-naïve patients achieved a median PFS of 8.36 months, consistent with these reports. Therefore, data supporting the combination in ICI-pretreated patients remains limited, particularly given the ongoing debate regarding ICI rechallenge in solid tumors [21]. Real-world retrospective evidence suggests that ICI rechallenge after a ≥12-month interval from prior ICI may yield improved outcomes in aUC, with optimal sequencing of anti-programmed cell death protein 1 (PD-1) after anti-PD-L1 achieving an OS of 26.4 months [22]. In this study, we did not exclude patients who had received prior ICI-based therapies. As anticipated, ICI-pretreated patients exhibited inferior outcomes. They achieved a PFS of 4.07 months and an OS of 13.19 months; none of them achieved CR. Nevertheless, the combination still provided PFS and OS advantages compared with previous second-line chemotherapy trials [2,17]. These results suggest that ICI rechallenge combined with nab-paclitaxel is a viable option for pretreated aUC.

Although ICIs have demonstrated substantial efficacy in aUC, reliable biomarkers for predicting response remain limited. For instance, PD-L1 expression on tumor cells or the combined positive score has proven to be effective biomarkers for forecasting ICI efficacy in various solid tumors, such as non-small cell lung cancer and melanoma. In the present study, we found no statistically significant associations between PD-L1 expression and clinical outcomes, and patients with PD-L1 <1% appeared to have a 10-month advantage in OS. However, the number of patients with available PD-L1 expression data was limited, and only 5 patients had PD-L1 expression ≥ 1%, which may have compromised the results. By contrast, data from large phase III trials and meta-analyses in aUC have shown that PD-L1 expression is associated with improved ORR, PFS, and OS following ICI administration [23–26]. However, the predictive value of PD-L1 remains controversial, with evidence suggesting that it is unlikely to serve as a standalone biomarker for selecting patients who will respond to ICIs [26]. Conflicting results across studies may stem from assay variability, such as differences between SP142 and 22C3 immunohistochemistry methods, which can yield discordant PD-L1 phenotypes and influence clinical outcomes [27]. Consequently, further investigation into more reliable biomarkers is warranted.

In recent years, emerging evidence indicates that genomic alterations could affect the efficacy of ICI treatment [28]. In this study, alterations in *PIK3C2B* and *PIK3CA* were observed exclusively among responders. Prior research has linked activation of the phosphoinositide 3-kinase pathway to enhanced benefits from immunotherapy in colorectal cancer, potentially owing to increased intratumoral infiltration of cytotoxic T cells [29]. Conversely, nonresponders are more likely to harbor epigenetic alterations in genes such as *KMT2A* and *ARID1A*. Recent studies have shown that alterations in *ARID1A*, particularly loss-of-function mutations, may be associated with favorable responses across various malignancies. Translational studies indicated that *ARID1A* dysfunction could activate the cyclic GMP-AMP synthase-stimulator of interferon genes (cGAS-STING) pathway in a DNA damage repair deficiency manner, thereby augmenting cytotoxic T-cell function [30,31]. Unfortunately, current evidence remains insufficient to support the use of single-gene alterations as reliable predictors of ICI efficacy in aUC. More comprehensive explorations, such as integrating gene signatures and novel algorithms, are needed to identify robust biomarkers.

Despite the potential of genomic profiling in stratifying patients who might benefit from ICI-based treatment, its routine application in clinical practice remains challenging. Tumor samples may be insufficient for sequencing, costs are unaffordable for uncovered insurance, and sequencing is time-consuming, often delaying clinical decisions. Alternatively, pretreatment serum levels of cytokines, chemokines, growth factors, and other soluble proteins may serve as practical biomarkers. In this study, we measured the concentrations of 28 serum biomarkers before treatment with camrelizumab combined with nab-paclitaxel. We found that elevated pretreatment serum levels of IL-6, IGFBP2, and CD25 were significantly associated with worse outcomes, because a subset of patients had received prior ICI, which may have durably altered the pretreatment serum cytokine profile through persistent immunomodulatory effects or changes in the tumor microenvironment. By stratifying patients into ICI-pretreated and ICI-naïve, we found that high levels of CD25 and IL-6 remained significantly associated with worse outcomes in ICI-naïve patients. Since only 2 responders were ICI-pretreated, no statistically significant associations were observed among ICI-pretreated patients, likely due to limited statistical power. A study showed that IL-6 can bind to gp130, thereby promoting tumor growth and suppressing the function of T cells activated by myeloid cells via signal transducer and activator of transcription 3 signaling [32]. IGFBP2, a member of the IGFBP family, maintains the serum level of insulin-like growth factor 1. In addition, other IGFBPs were higher in nonresponders, though they did not cross the statistical boundary. Several studies showed that an imbalance of insulin-like growth factor 1 was attributed to tumor progression and immune evasion [33,34]. CD25, also known as interleukin-2 receptor subunit alpha (IL-2R), is the ligand for interleukin-2 (IL-2) on the cell membrane. The activation of IL-2R is the marker of cytotoxic T-cell proficiency. However, the CD25 we measured was not on the membrane, which shares the same ligand, potentially leading to insufficient T-cell activation. Previous studies have shown that elevated serum levels of CD25 are associated with worse outcomes in patients receiving immunotherapy [35,36]. Collectively, these findings suggest that pretreatment serum levels of IL-6, IGFBPs, and CD25 may serve as surrogate biomarkers for predicting the efficacy of camrelizumab plus nab-paclitaxel.

We acknowledge that the present study has several limitations. First, this was a single-arm trial without a randomized control group, which precludes direct comparison with standard-of-care treatments. Although the observed outcomes appear promising relative to historical controls, a phase 3 randomized controlled trial is required to confirm efficacy and establish the role of this regimen in clinical practice. Second, data on established tumor biomarkers, such as PD-L1 expression, genomic profiling, and pretreatment serum biomarker levels, were limited, warranting further validation. Third, the number of ICI-pretreated patients was small, which likely confounded the interpretation of the cytokine data; a more comprehensive investigation is needed. Finally, although the study met its prespecified statistical enrollment target, the overall sample size remained relatively small. This limits the power to detect subgroup effects or perform multivariable adjustments. Larger, multicenter trials are essential to validate these preliminary observations.

In summary, we present the results of camrelizumab in combination with nab-paclitaxel for previously treated aUC patients, which showed modest antitumor activity and acceptable tolerability.

## Ethics Approval

This clinical study was approved by the ethics committee of Sun Yat-sen University Cancer Center (Approval number: B2020-041) and the ethics committee of the Second Affiliated Hospital of Kunming Medical University (Approval number: PJ-2021-87). All procedures performed in this study involving human participants were in accordance with the ethical standards of the institutional research committee and with the Helsinki Declaration. Written informed consent was obtained from all participants prior to their enrollment in the study.

## Data Availability

The data collected and used in this study are available from the corresponding authors upon reasonable request. The authenticity of this manuscript has been validated by uploading the key raw data to the Research Data Deposit public platform (www.researchdata.org.cn) under approval RDD number RDDA2026446753.

## Supplementary Materials

Supplementary 1 Tables S1 to S3 Figs. S1 to S5 CONSORT checklist
