# Supplementary material for: Camrelizumab Plus Nab-paclitaxel in Patients with Previously Treated Advanced Urothelial Carcinoma: A Multicenter Phase II Study
Source: Cancer Commun (Lond). 2026 Apr 21;46:0025. doi: 10.34133/cancomm.0025 (PMC13096681; doi:10.34133/cancomm.0025)
Supplement: Supplementary 1 — Tables S1 to S3 Figs. S1 to S5 CONSORT checklist [file cancomm.0025.f1.zip › CANCOMM-D-25-00285-R4-Supplementary-marked.docx]

**Supplementary Materials for**

**Camrelizumab Plus Nab-paclitaxel in Patients with Previously Treated Advanced Urothelial Carcinoma: A Multicentre Phase II Study**

**Running title:** Camrelizumab plus nab-paclitaxel in advanced urothelial carcinoma.

**Authors:** Haifeng Li^1, 2, †^, Meiting Chen^1, 2, †^, Riqing Huang^1, 2, †^, Qixiang Rong^1, 2, †^, Jing Hao^1, 3^, Qiufan Zheng^1, 2^, Yanhong Su^1, 2^, Ditian Shu^1, 2^, Yue Zhang^1, 2^, Wei Yang^1, 2^, Xuefen Lei^4^, Yuchen Cai^1^, Cong Xue^1, 2^, Xin An^1, 2, *^, Yanxia Shi^1,2, *^

**Affiliations:**

^1^ State Key Laboratory of Oncology in South China, Guangdong Provincial Clinical Research Center for Cancer, Sun Yat-sen University Cancer Center, Guangzhou 510060, P. R. China.

^2^ Department of Medical Oncology, Sun Yat-sen University Cancer Center, Guangzhou 510060, P. R. China.

^3^ Zhongshan School of Medicine, Sun Yat-sen University, Guangzhou 510060, P. R. China.

^4^ Department of Medical Oncology, The Second Affiliated Hospital of Kunming Medical University, Kunming 650101, P. R. China.

^*^**Corresponding author**

1. Yanxia Shi, Department of Medical Oncology, Sun Yat-sen University Cancer Center, 651 Dongfeng Road East 651, Guangzhou 510060, Guangdong, P. R. China

Email address: shiyx@sysucc.org.cn

2. Xin An, Department of Medical Oncology, Sun Yat-sen University Cancer Center, 651 Dongfeng Road East, Guangzhou 510060, Guangdong, P. R. China

Email address: anxin@sysucc.org.cn

^†^Haifeng Li, Meiting Chen, Riqing Huang, and Qixiang Rong contributed equally to this study.

**Table S1. Targeted gene sequencing panel**


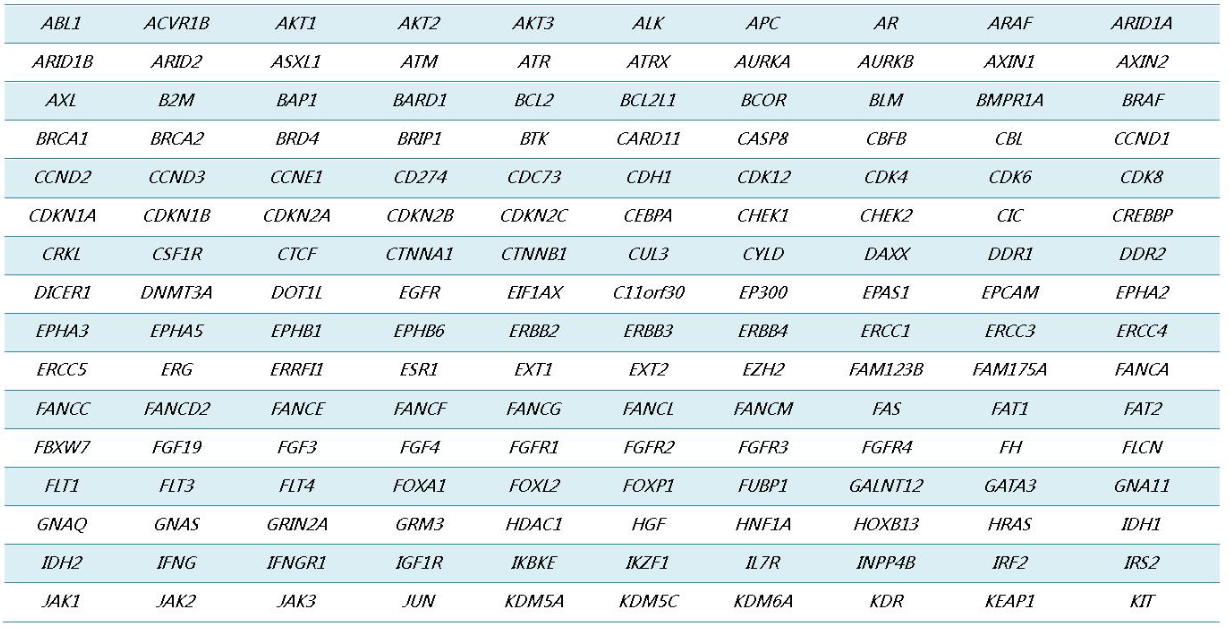

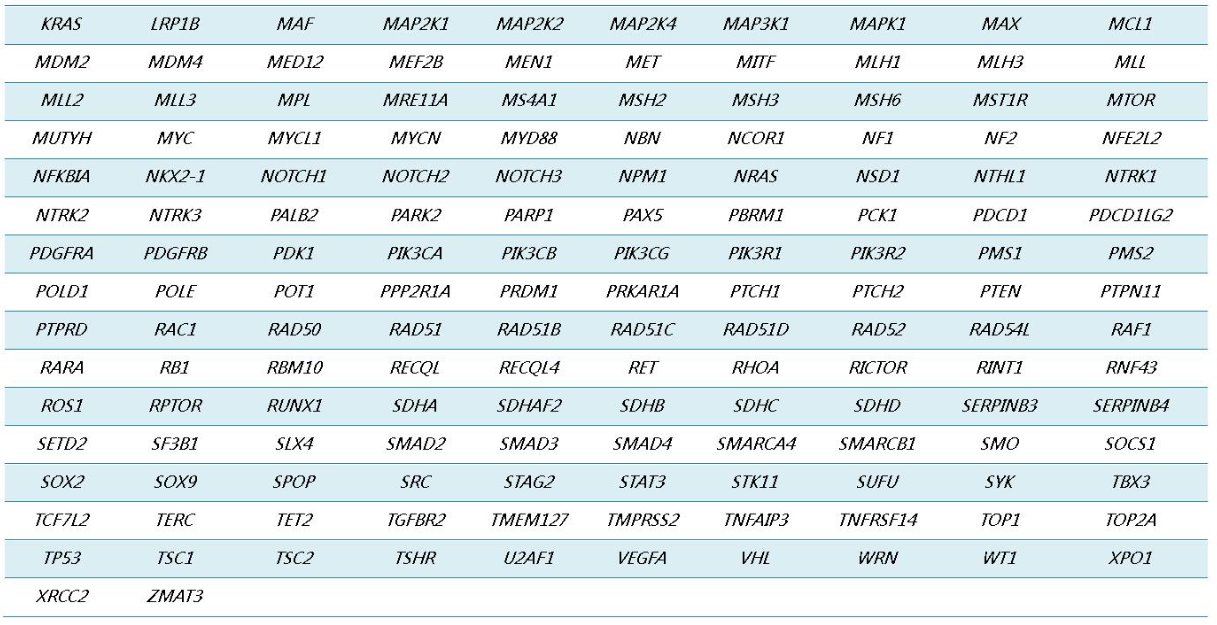

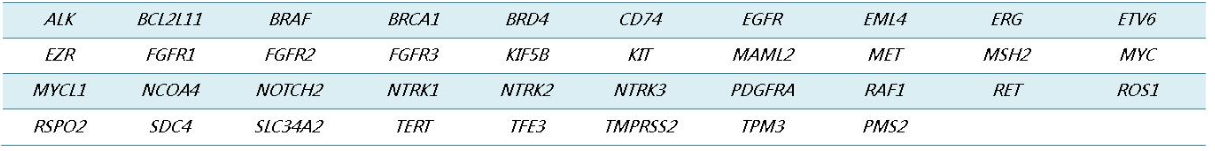

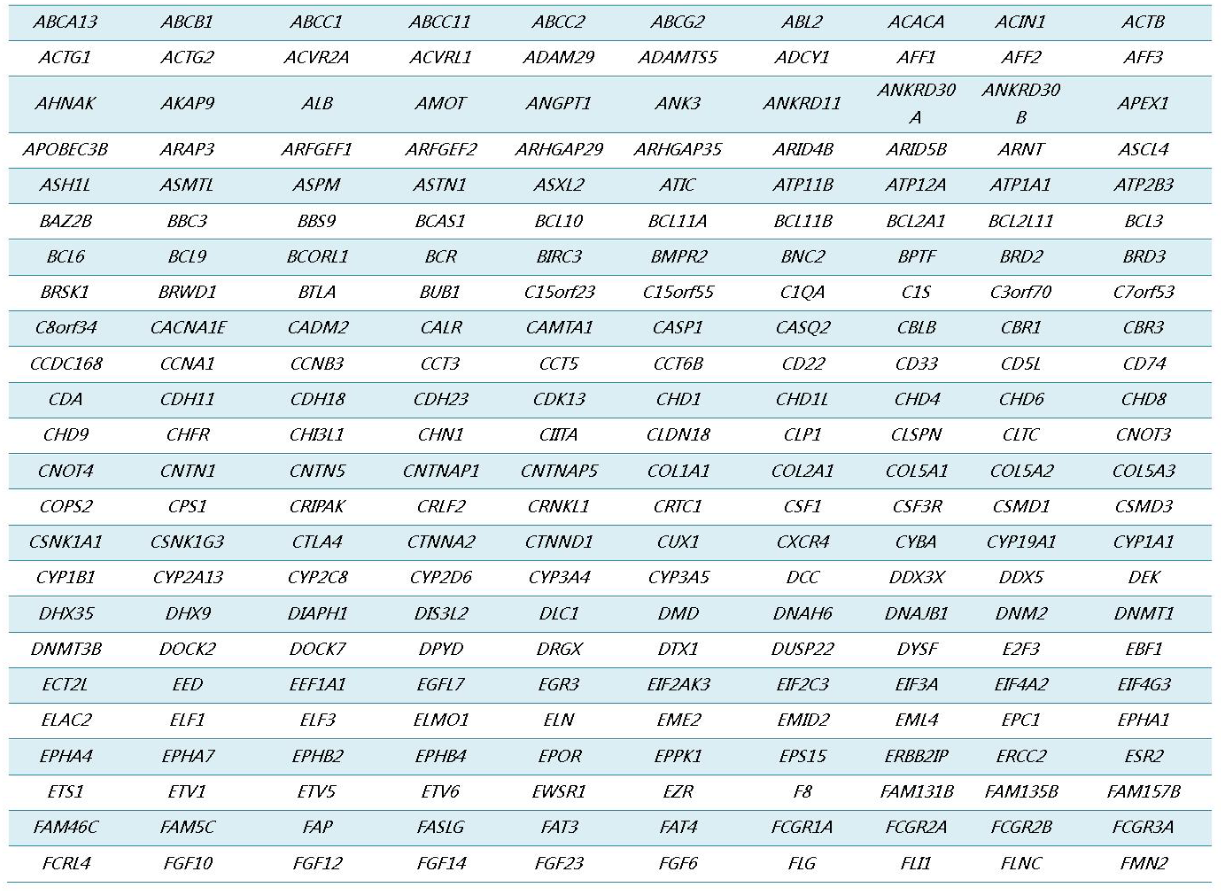

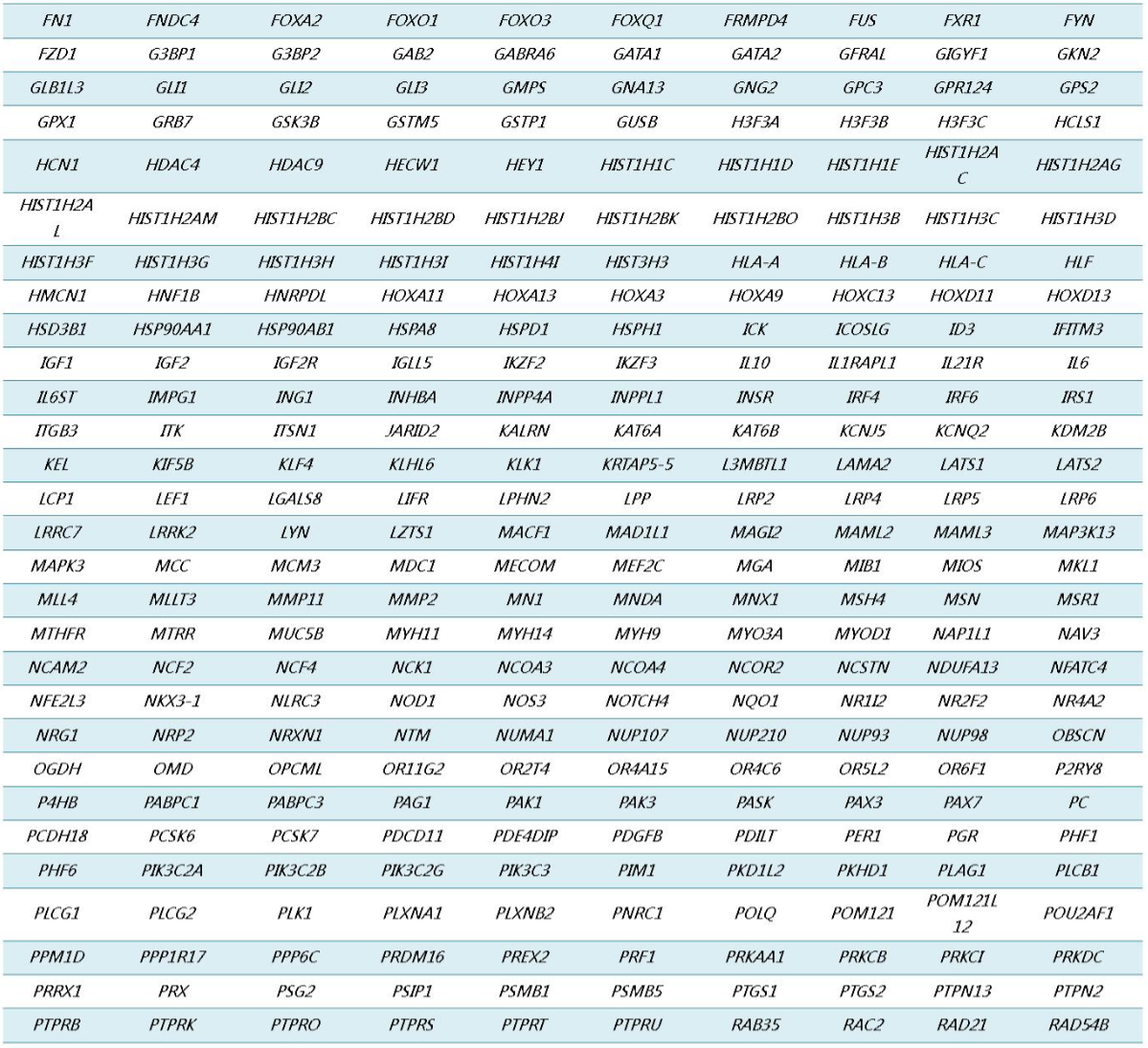

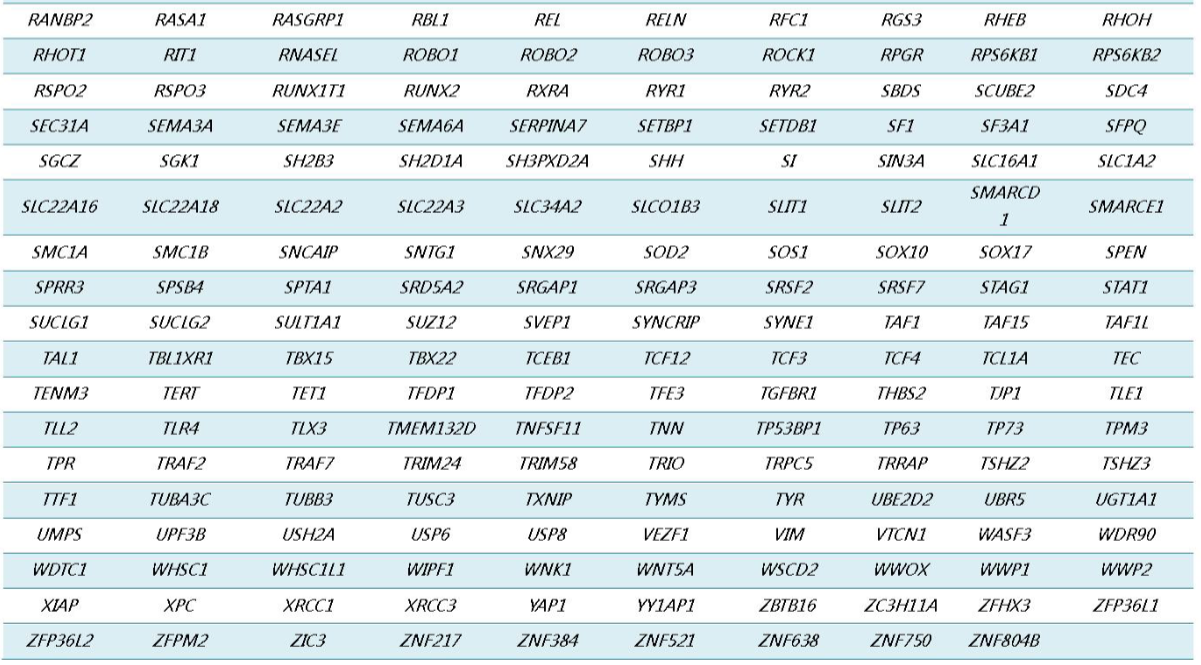

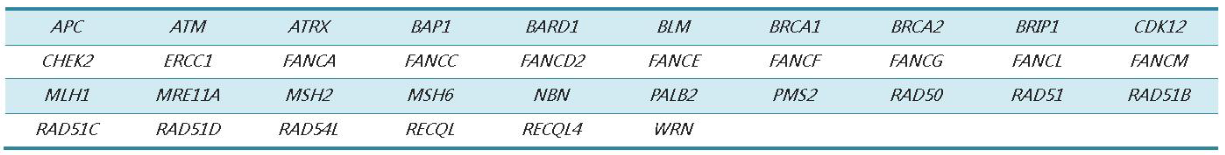


**Table S2. Treatment responses based on RECIST 1.1 in the PPS population (*n* = 49)**

| **Characteristics** | ***n*** | **CR** | **PR** | **SD** | **PD** | ***P* value** |
| --- | --- | --- | --- | --- | --- | --- |
| **Total** | 49 | 4 (8.16%) | 16 (32.65%) | 16 (32.65%) | 13 (26.54%) |  |
| **Age** | | | | | | 0.125 |
| <65 | 25 | 4 (16.00%) | 6 (24.00%) | 7 (28.00%) | 8 (32.00%) |  |
| ≥65 | 24 | 0 (0.00%) | 10 (41.67%) | 9 (37.50%) | 5 (20.83%) |  |
| **Sex** | | | | | | 0.188 |
| Female | 15 | 3 (20.00%) | 5 (33.33%) | 5 (33.33%) | 2 (13.33%) |  |
| Male | 34 | 1 (2.94%) | 11 (32.35%) | 11 (32.35%) | 11 (32.35%) |  |
| **ECOG PS** | | | | | | 0.479 |
| 0 | 14 | 2 (14.29%) | 7 (50.00%) | 2 (14.29%) | 3 (21.43%) |  |
| 1 | 30 | 2 (6.67%) | 8 (26.67%) | 12 (40.00%) | 8 (26.67%) |  |
| 2 | 5 | 0 (0.00%) | 1 (20.00%) | 2 (40.00%) | 2 (40.00%) |  |
| **Site of primary tumor** | | | | | | 0.710 |
| BUC | 13 | 1 (7.69%) | 3 (23.08%) | 6 (46.15%) | 3 (23.08%) |  |
| UTUC | 36 | 3 (8.33%) | 13 (36.11%) | 10 (27.78%) | 10 (27.78%) |  |
| **Metastases** | | | | | | 1.000 |
| Locally advanced | 2 | 0 (0.00%) | 1 (50.00%) | 1 (50.00%) | 0 (0.00%) |  |
| Visceral disease | 47 | 4 (8.51%) | 15 (31.91%) | 15 (31.91%) | 13 (27.66%) |  |
| **Site of metastases** | | | | | | 0.970 |
| Lymph node only | 13 | 1 (7.69%) | 4 (30.77%) | 3 (23.08%) | 5 (38.46%) |  |
| Liver metastases | 3 | 0 (0.00%) | 1 (33.33%) | 2 (66.67%) | 0 (0.00%) |  |
| Lung metastases | 7 | 1 (14.29%) | 2 (28.57%) | 3 (42.86%) | 1 (14.29%) |  |
| Peritoneal metastases | 22 | 2 (9.09%) | 7 (31.82%) | 7 (31.82%) | 6 (27.27%) |  |
| Multi visceral metastases | 2 | 0 (0.00%) | 1 (50.00%) | 0 (0.00%) | 1 (50.00%) |  |
| **PD-L1 status** | | | | | | 0.075 |
| <1% | 22 | 0 (0.00%) | 4 (18.18%) | 10 (45.45%) | 8 (36.36%) |  |
| ≥1% | 4 | 0 (0.00%) | 3 (75.00%) | 1 (25.00%) | 0 (0.00%) |  |
| **Number of previous systemic regimens** | | | | | | 0.275 |
| 1 | 40 | 4 (10.00%) | 12 (30.00%) | 15 (37.50%) | 9 (22.50%) |  |
| >1 | 9 | 0 (0.00%) | 4 (44.44%) | 1 (11.11%) | 4 (44.44%) |  |
| **Previous ICI-based therapy** | | | | | | 0.018 |
| No | 36 | 4 (11.11%) | 13 (36.11%) | 14 (38.89%) | 5 (13.89%) |  |
| Yes | 13 | 0 (0.00%) | 3 (23.08%) | 2 (15.38%) | 8 (61.54%) |  |

Abbreviations: BUC, bladder urothelial carcinoma; CR, complete response; DCR, disease control rate; ECOG PS, Eastern Cooperative Oncology Group performance status; ICI, immune checkpoint inhibitor; NOS, not otherwise specified; PPS, per protocol set; PD, progressive disease; PD-L1, Programmed cell death ligand 1; PR, partial response; RECIST 1.1, Response Evaluation Criteria in Solid Tumors version 1.1; SD, stable disease; UTUC, upper tract urothelial carcinoma.

**Table S3. The differences in pretreatment serum biomarker levels (pg/ml).**

| Biomarkers | PFS ≥6 mo. and ICI naïve  (*n* = 15) | PFS <6 mo. and ICI naïve  (*n* = 8) | *P* value | PFS ≥6 mo. and ICI pretreated  (*n* = 2) | PFS <6 mo. and ICI pretreated  (*n* = 7) | *P* value |
| --- | --- | --- | --- | --- | --- | --- |
| CCL2 | 563.95 | 298.92 | 1.000 | 197.80 | 229.91 | 0.462 |
| CD25 | 885.39 | 1,671.28 | 0.012 | 1,320.50 | 1,600.86 | 0.462 |
| CXCL10 | 45.62 | 67.68 | 0.220 | 122.50 | 95.57 | 0.889 |
| CXCL11 | 84.95 | 122.83 | 0.041 | 118.38 | 108.70 | 0.769 |
| CXCL13 | 107.27 | 204.54 | 0.093 | 156.02 | 167.99 | 0.303 |
| CXCL2 | 389.86 | 544.18 | 1.000 | 204.84 | 733.06 | 0.889 |
| CXCL9 | 1,386.17 | 1,799.38 | 0.006 | 2,002.50 | 1,474.79 | 0.238 |
| FGF2 | 8.93 | 12.84 | 0.207 | 12.71 | 10.93 | 0.659 |
| IFNa | 8.37 | 8.86 | 0.697 | 8.31 | 10.26 | 0.238 |
| IFNb | 9.22 | 11.25 | 0.134 | 10.96 | 9.88 | 0.722 |
| IFNg | 7.10 | 5.75 | 0.435 | 7.66 | 10.03 | 0.883 |
| IGFBP1 | 24,344.33 | 56,378.00 | 0.017 | 81,620.00 | 30,308.46 | 0.106 |
| IGFBP2 | 203,958.07 | 332,502.13 | 0.001 | 378,110.00 | 237,200.29 | 0.111 |
| IGFBP3 | 417,229.34 | 552,499.50 | 0.076 | 425,131.50 | 512,756.86 | 0.500 |
| IGFBP4 | 238.58 | 240.80 | 0.922 | 275.20 | 250.22 | 0.883 |
| IGFBP6 | 30,390.87 | 30,885.50 | 0.548 | 30,593.00 | 30,273.71 | 1.000 |
| IGFBP7 | 13,728.27 | 17,857.13 | 0.231 | 17,767.50 | 15,240.86 | 0.462 |
| IL10 | 8.05 | 4.80 | 0.497 | 3.72 | 6.37 | 0.667 |
| IL17 | 9.32 | 10.15 | 0.283 | 10.33 | 9.58 | 0.623 |
| IL1a | 25.97 | 26.89 | 0.266 | 27.72 | 27.48 | 1.000 |
| IL1b | 16.05 | 16.92 | 0.275 | 17.48 | 16.27 | 0.361 |
| IL2 | 13.82 | 14.01 | 0.472 | 14.94 | 13.53 | 0.280 |
| IL4 | 41.31 | 54.69 | 0.026 | 63.50 | 40.29 | 0.234 |
| IL6 | 5.47 | 21.81 | 0.002 | 14.35 | 6.49 | 0.768 |
| MMP10 | 2,215.28 | 2,503.38 | 0.040 | 1,972.50 | 1,778.43 | 0.557 |
| MMP7 | 3,933.27 | 5,453.00 | 0.466 | 3,337.21 | 4,489.57 | 0.667 |
| VEGFA | 254.02 | 254.56 | 0.675 | 59.49 | 201.38 | 0.142 |
| VEGFC | 422.33 | 401.07 | 1.000 | 258.16 | 554.30 | 0.056 |

Abbreviations: CCL, C-C motif chemokine ligand; CD, cluster of differentiation; CXCL, C-X-C motif chemokine ligand; FGF, fibroblast growth factor; ICI, immune checkpoint inhibitor; IFN, interferon; IGFBP, insulin-like growth factor-binding protein; IL, interleukin; MMP, matrix metalloproteinase; PFS, Progression-free survival; VEGF, vascular endothelial growth factor.


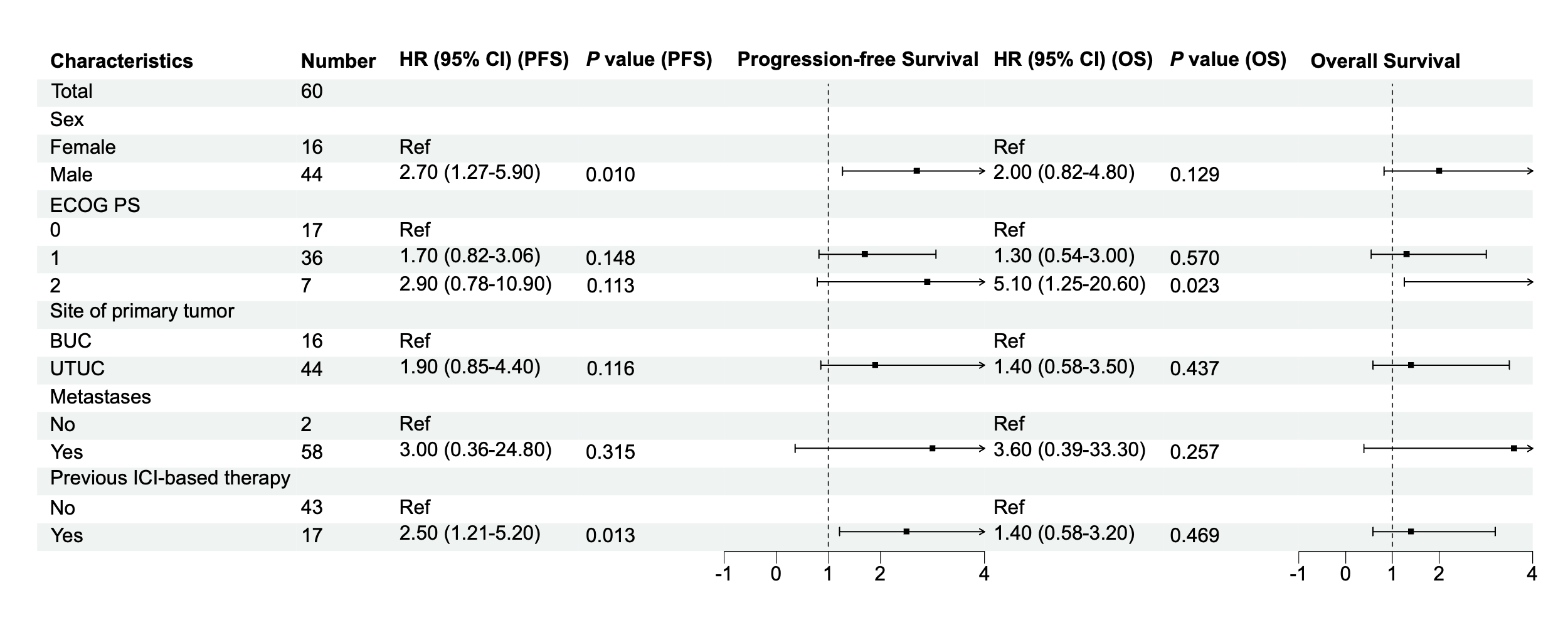
**Figure S1. Multivariable Cox analyses for FAS.** Survival analyses were performed using multivariable Cox regression. Abbreviations: BUC, bladder urothelial carcinoma; CI, confidence interval; ECOG PS, Eastern Cooperative Oncology Group Performance Status; FAS, full analysis set; HR, hazard ratio; NR, not reached; ICI, immune checkpoint inhibitor; PFS, Progression-free survival; Ref, reference; OS, overall survival; UTUC, upper tract urothelial carcinoma.


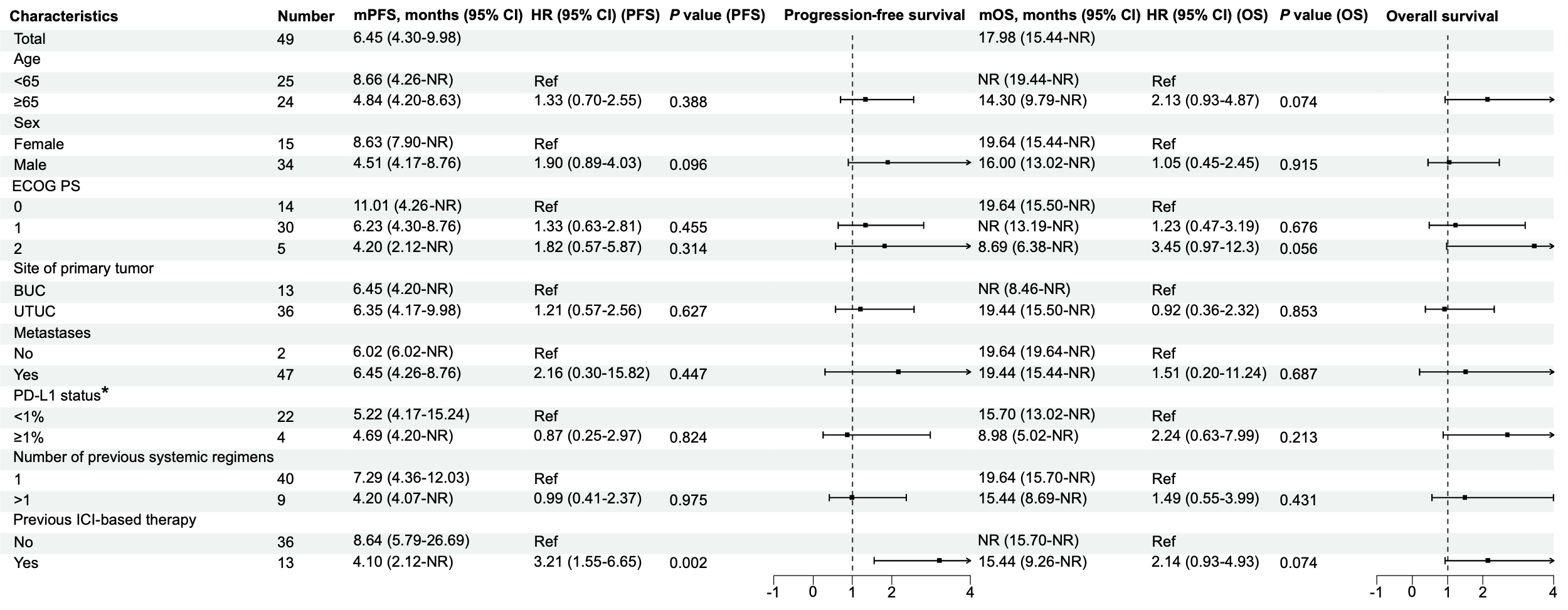


**Supplementary Figure S2. Subgroup analyses of PFS and OS for the PPS population (*n* = 49).** Survival analyses were performed using univariable Cox regression. ^*^There are 23 patients with unknown PD-L1 status. Abbreviations: BUC, bladder urothelial carcinoma; CI, confidence interval; ECOG PS, Eastern Cooperative Oncology Group Performance Status; PD-L1, Programmed cell death ligand 1; PPS, per protocol set; HR, hazard ratio; NR, not reached; ICI, immune checkpoint inhibitor; mPFS, median progression-free survival; Ref, reference; mOS, median overall survival; UTUC, upper tract urothelial carcinoma.


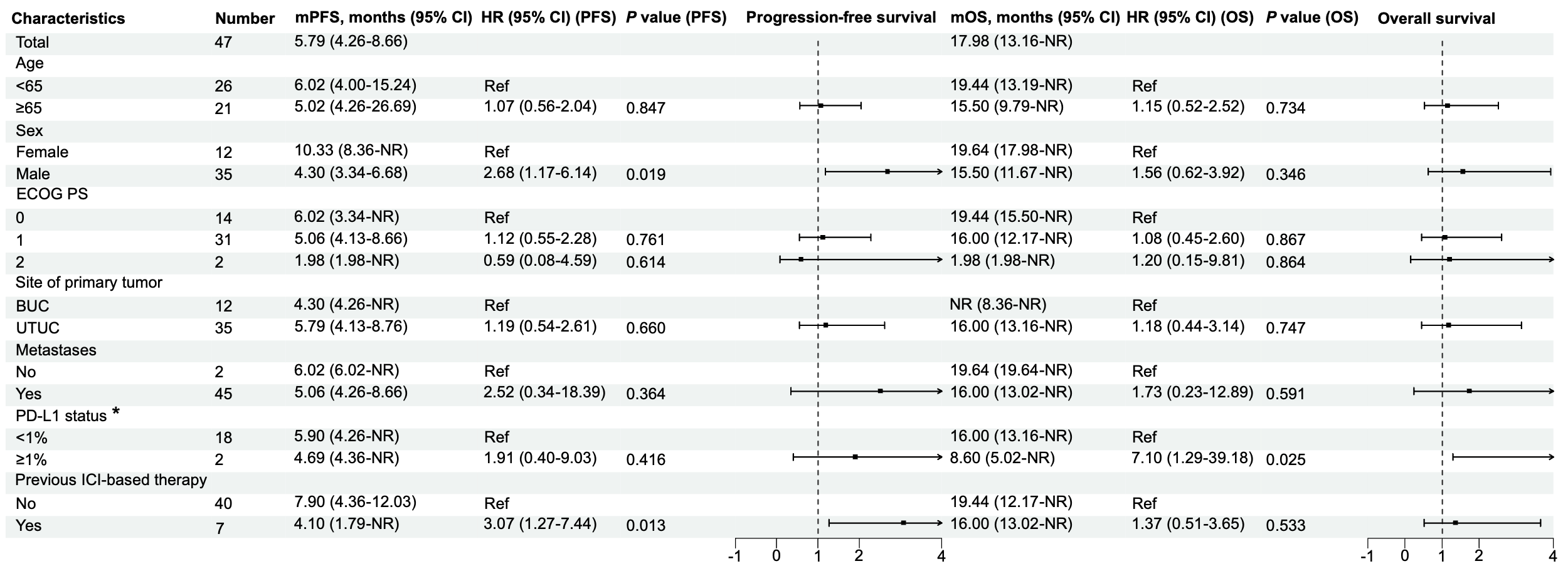


**Supplementary Figure S3. Subgroup analyses of PFS and OS in patients treated in the second-line setting (*n* = 47).** Survival analyses were performed using univariable Cox regression.^*^There are 27 patients with unknown PD-L1 status. Abbreviations: BUC, bladder urothelial carcinoma; CI, confidence interval; ECOG PS, Eastern Cooperative Oncology Group Performance Status; HR, hazard ratio; NR, not reached; ICI, immune checkpoint inhibitor; mPFS, median progression-free survival; PD-L1, Programmed cell death ligand 1; Ref, reference; mOS, median overall survival; UTUC, upper tract urothelial carcinoma.


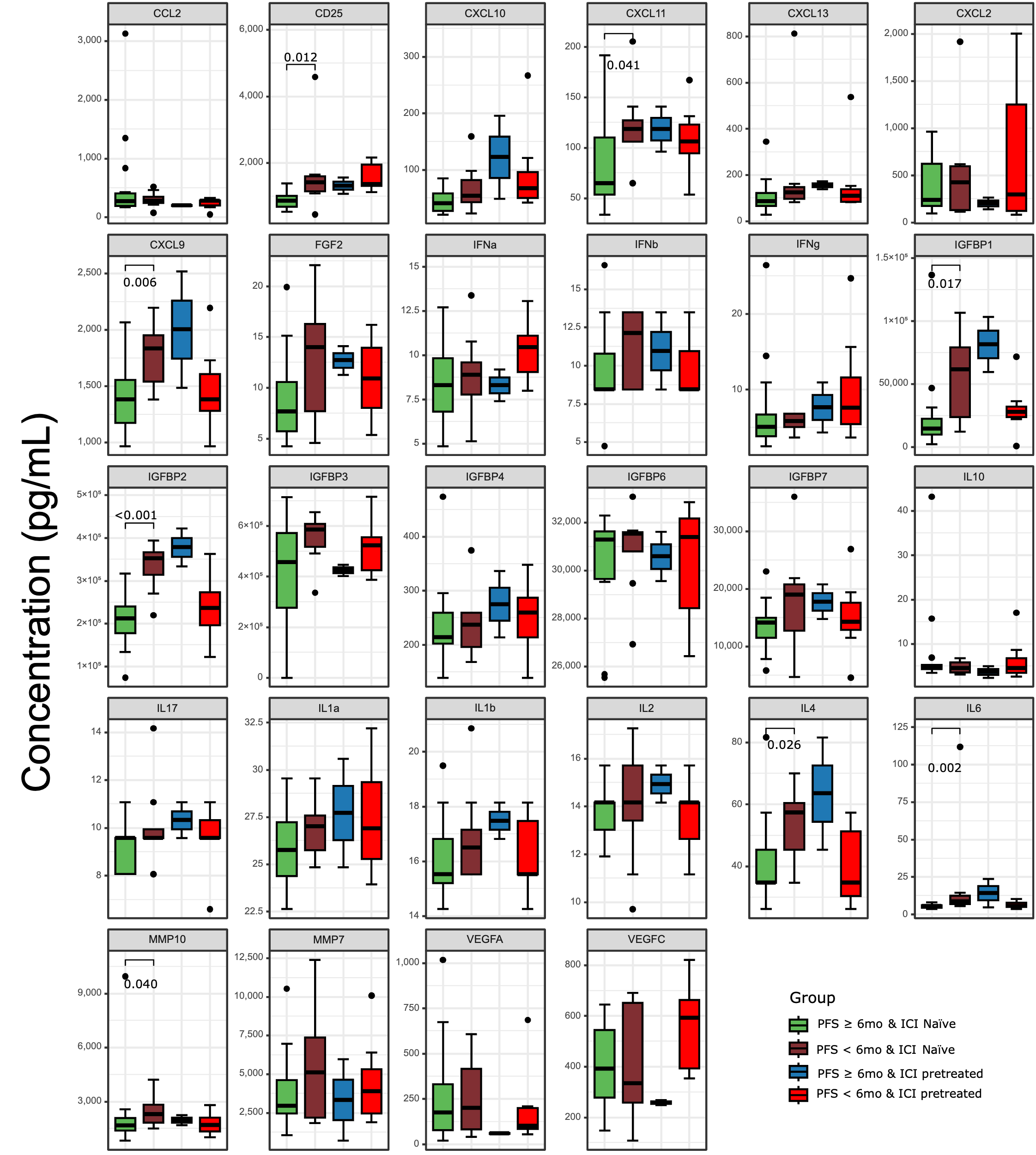


**Supplementary Figure S4. Biomarker analyses of pretreatment serum levels of biomarker stratified by prior ICI treatment.** Differences in pretreatment serum cytokine levels between responders (PFS ≥6 months) and non-responders (PFS <6 months), stratified by prior ICI treatment, were assessed using the Wilcoxon test to evaluate statistical significance. Abbreviations: CCL, C-C motif chemokine ligand; CD, cluster of differentiation; CXCL, C-X-C motif chemokine ligand; FGF, fibroblast growth factor; ICI, immune checkpoint inhibitor; IFN, interferon; IGFBP, insulin-like growth factor-binding protein; IL, interleukin; MMP, matrix metalloproteinase; PFS, Progression-free survival; VEGF, vascular endothelial growth factor.

**
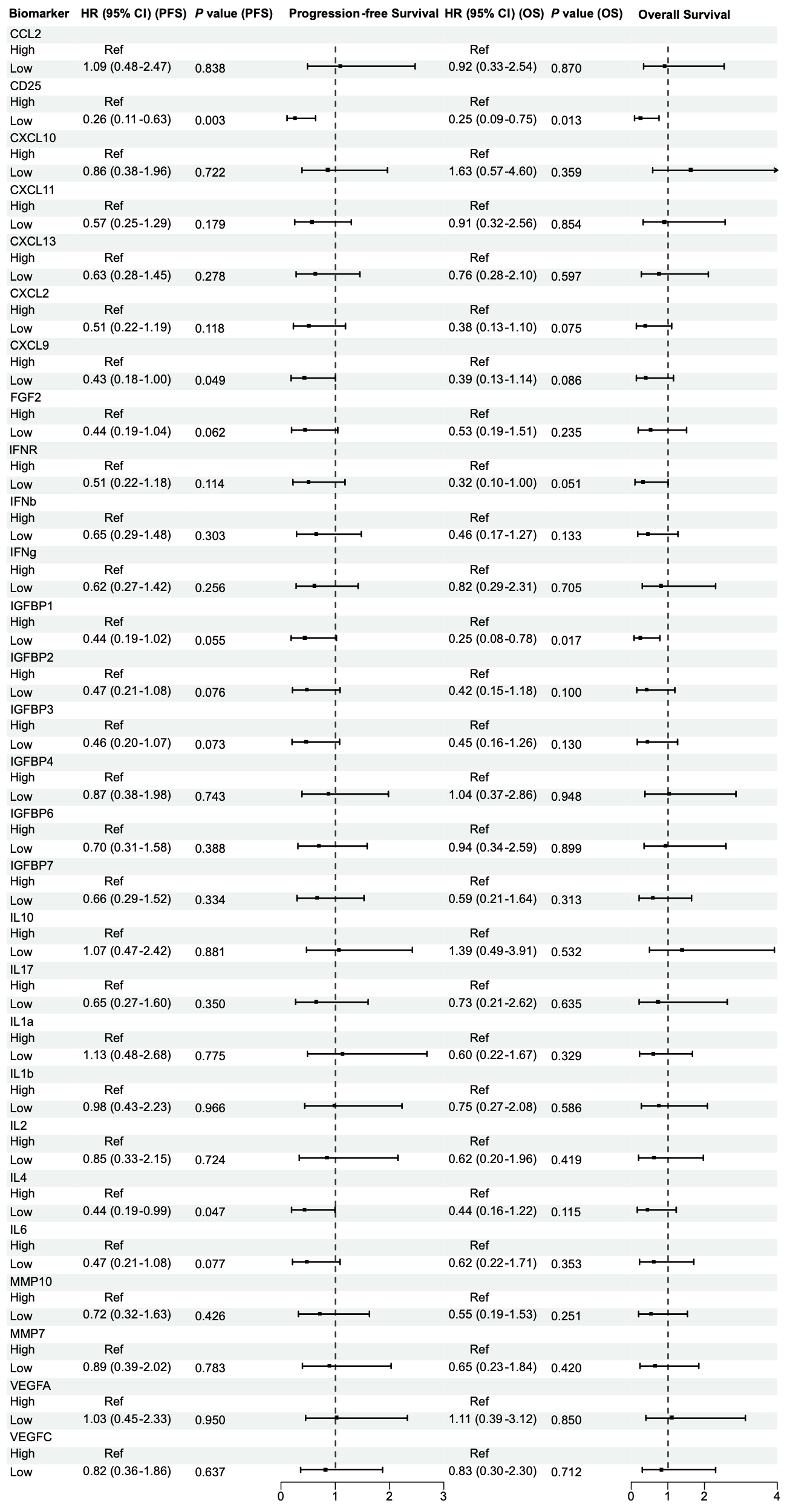
**

**Supplementary Figure S5. Survival analyses of PFS and OS for serum biomarkers.** Survival analyses were performed using univariable Cox regression, with the cutoff point set at the median pretreatment serum cytokine level. Abbreviations: CCL, C-C motif chemokine ligand; CD, cluster of differentiation; CI, confidence interval; CXCL, C-X-C motif chemokine ligand; FGF, fibroblast growth factor; HR, hazard ratio; ICI, immune checkpoint inhibitor; IFN, interferon; IGFBP, insulin-like growth factor-binding protein; IL, interleukin; MMP, matrix metalloproteinase; OS, overall survival; PFS, Progression-free survival; Ref, reference; VEGF, vascular endothelial growth factor.
